# Impact of metal density on deformity correction in posterior fusions for adolescent idiopathic scoliosis: A retrospective cohort study

**DOI:** 10.1016/j.amsu.2020.02.011

**Published:** 2020-03-10

**Authors:** Irfan Qadir, Abdullah Shah, Syed Roman Alam, Haseeb Hussain, Rizwan Akram, Amer Aziz

**Affiliations:** Department of Orthopaedics and Spine Surgery, Ghurki Trust Teaching Hospital, Lahore, Pakistan

**Keywords:** Adolescent idiopathic scoliosis, Posterior instrumentation, Metal density, Correction, Pakistan

## Abstract

**Introduction:**

Optimal implant density for posterior spinal fusion in adolescent idiopathic scoliosis (AIS) remains controversial. We aimed to examine radiographic outcomes of AIS cases treated with limited density pedicle screw constructs.

**Methods:**

This is a retrospective analysis of 96 patients (89 females and 7 males with mean age of 13.8 ± 4.4 years) with AIS who underwent posterior spinal instrumentation at Ghurki Trust Teaching Hospital between 2014 and 2016. Construct characteristics and radiographic measurements were compared preoperatively and at 2 year follow-up using paired t-test. Pearson's correlation coefficient between curve characteristics and metal density was calculated.

**Results:**

Preoperative coronal Cobb angle was 68.5 ± 6.9°. Flexibility of the curve was 47.5 ± 10.3% based on push-prone films. The mean number of vertebrae in the fusion was 10.7 ± 1.6. The implant density was 62%. The mean postoperative Cobb angle was 18.6 ± 4.2°, giving a mean correction of 72.5 ± 6.8%. Metal density was not correlated with preoperative coronal or sagittal radiographic variables; MT Cobb angle (r = 0.02, p = 0.847), MT curve flexibility (r = 0.129, p = 0.210), preoperative thoracic kyphosis (r = -0.119, p = 0.247) or lumbosacral lordosis (r = −0.048, p = 0.645). There was a significant correlation between the flexibility of the curve as assessed by push-prone radiographs with the percentage correction achieved (r = 0.368, p < 0.0001) as well as absolute correction in degrees (r = 0.643, p < 0.0001). No significant correlations were present between metal density and MT curve coronal correction rate/percentage (r = 0.086, p = 0.407) or postoperative Cobb angle (r = 0.098, p = 0.344).

**Conclusion:**

Metal density does not influence the coronal and sagittal correction of AIS. Neither larger nor stiffer curves necessitate high metal density.

**Level of evidence:**

IV.

## Highlights

• Metal density does not influence either amount of main thoracic curve correction or post-operative Cobb angle.
• We conclude that posterior fusion constructs with implant densities of approximately 60% can safely be used in moderate, flexible (45–80°, average 47% flexibility) idiopathic curves to attain and maintain correction in adolescent idiopathic scoliosis.
• There is a significant correlation between the flexibility of the curve as assessed by push-prone radiographs with the percentage correction achieved as well as absolute correction in degrees.
• We found that preoperative coronal or sagittal radiographic variables like main thoracic Cobb angle, curve flexibility, preoperative thoracic kyphosis or lumbosacral lordosis did not influence surgeon's decision for implant density.
• Longer fusions were required for larger and less flexible curves.

## 1 Introduction

Posterior spinal fusion with pedicle screws has become the “gold standard” for the management of adolescent idiopathic scoliosis (AIS) [1]. Pedicle screws provide three column fixation through the strongest part of vertebra thereby enhancing surgeon's ability to do a 3-dimensional deformity correction. The higher pullout strength results in less long-term loss of correction, shorter fusions resulting in preservation of motion segments, lower pseudarthrosis rates and lower implant failures compared with these alternative posterior instrumentation systems [[2], [3], [4]].However, studies are contradictory regarding the effect of metal density on coronal and sagittal curve corrections [[5], [6], [7], [8], [9], [10], [11], [12]]. Multiple factors including curve flexibility, instrumentation and rod types, reduction strategies and curve types, affect outcomes [[13], [14], [15], [16], [17]]. Given this, intraoperative decisions regarding the number of anchorage points remain difficult, with considerable inter-surgeon variability [18,19].

The rationale for using a high implant density constructs (i.e. instrumenting every available pedicle) is to obtain more rigid fixation and to limit potential stress concentration at any one screw [4]. Furthermore, health-related quality of life instruments such as the SRS 22, 24, or 30 seem to show little correlation with curve correction [2,6,20,21]. The placement of every additional pedicle screw is associated with increased operative time, bleeding, risk of neurological deterioration and increased implant cost [22]. If implant density can be lowered without compromising clinical results, reducing the number of screws may improve the efficiency and cost effectiveness of scoliosis surgery [1]. Several authors have demonstrated successful results with low-density instrumentation for the treatment of scoliosis [10,11,[23], [24], [25], [26]]. The purpose of this retrospective review is to examine the radiographic outcomes of AIS cases treated with limited density pedicle screw constructs.

### 1.1 Patients and methods

After institutional review board approval, retrospective analysis of 96 consecutive patients with adolescent idiopathic scoliosis (AIS) who underwent posterior spinal instrumentation at Ghurki Trust Teaching Hospital between January 2014 and December 2016 was performed. Patients with AIS were included in the study if aged more than 10 years at the time of fusion, with a fusion construct consisting of all pedicle screws except for bilateral hooks at the uppermost instrumented vertebra, and a major curve magnitude between 45 and 80°. Scoliosis patients with a diagnosis other than AIS, previous spine surgery, the use of additional spinal osteotomies, a Lenke 5 curve, patients with less than 2 years’ follow-up, or the use of hooks or wires below the uppermost instrumented level were excluded from the study. The study was conducted and reported in line with STROCSS criteria [27]. Research registration: NCT04175145 http://www.clinicaltrials.gov.

Same surgical team operated on all the patients (AS and AA). Spine was instrumented using the PolyNices Spine System (Kanghui Medical Innovation, Medtronics subsidiary, China). A fusion construct consisting of all pedicle screws except at uppermost instrumented vertebra was used in all patients. Contoured dual 6-mm titanium rods were used to connect the construct. Pedicle screw location and density was determined during preoperative planning by the surgeons (AS and AA) based on perceived curve stiffness and fusion length with an even distribution of fixation points along the construct. Pedicle screw instrumentation was performed by the free hand technique with biplanar fluoroscopic and direct screw EMG impedance testing for confirmation of placement. Reduction was via a cantilever-segmental-translation manoeuvre in all cases.

Radiographic parameters were measured and compared preoperatively, 2 weeks postoperatively, 2 years postoperatively, and at final follow-up. These included coronal Cobb angles, coronal best bend flexibility (%), thoracic kyphosis (T5-T12), lumbosacral lordosis (L1-S1) and Lenke classification. The implant density of each construct was calculated as number of pedicle screws used per pedicle available for fixation [number of screws used plus bilateral UIV hooks/(levels fused x 2)].Medical records were reviewed to obtain demographic information, operative time, estimated blood loss, and complications.

Data was entered and analyzed using SPSS v21. The distribution of the variables is given as the mean, standard deviation, and range. Bivariate analysis was performed using Pearson's correlation coefficient between radiographic variables [coronal plane curve preoperative Cobb angle, curve flexibility, percentage correction, CI for the main thoracic (MT) and lumbar curves; preoperative and absolute change in thoracic kyphosis T5-12 and lumbosacral lordosis (L1-S1)] and surgical variables [metal density, length of fusion]. The paired *t*-test was used to compare preoperative data with postoperative data. A *P* value of less than 0.05 was considered as being statistically significant.

## 2 Results

This study comprised of 96 patients including 89 females and 7 males with a mean age of 13.8 ± 4.4 years. Table 1 illustrates radiographic parameters of patients. Preoperatively, the deformity in the coronal plane as measured by Cobb angle of the end vertebrae on standing radiographs was 68.5 ± 6.9°. Flexibility of the curve was 47.5 ± 10.3% based on push-prone films. The mean number of vertebrae in the fusion was 10.7 ± 1.6. The implant density was 62% (range 56%–72%; median 64%). There was no correlation with preoperative MT coronal Cobb angle and preoperative thoracic kyphosis (r = 0.04, p = 0.687) or preoperative lumbosacral lordosis (r = −0.005, p = 0.965). Larger curves tended to be less flexible (r = −0.713, p < 0.001).

**Table 1:** Pre-operative and Post-operative radiographic Parameters.

	Mean ± SD
Major curve Cobb angle (degrees)	
Preoperative	68.5 ± 6.9
2 weeks post-operative	18.6 ± 4.2
2 years	22 ± 5.7
Best bend flexibility (%)	47.3 ± 10.5
Major curve Cobb correction (degrees)	
Initial	49.9 ± 8.3
2 years Followup	46.4 ± 8.6
Major curve Cobb correction (%)	
Initial	72.5 ± 6.8
2 years Followup	67.5 ± 8.9
Thoracic Kyphosis	
Preoperative	25.8 ± 6.8
2 weeks post-operative	21.6 ± 5.9
2 years	24.6 ± 6.3
Lumbar Lordosis	
Preoperative	52.5 ± 6.5
2 weeks post-operative	47.4 ± 5.2
2 years	50.4 ± 6.0
Absolute change in thoracic kyphosis (degrees)	
Initial	−4.2 ± 9.6
2 years	−1.2 ± 9.4
Absolute change in lumbar lordosis (degrees)	
Initial	−5.1 ± 8.2
2 years	−2.1 ± 8.7

Metal density was not correlated with preoperative coronal or sagittal radiographic variables; MT Cobb angle (r = 0.02, p = 0.847), MT curve flexibility (r = 0.129, p = 0.210), preoperative thoracic kyphosis (r = −0.119, p = 0.247) or lumbosacral lordosis (r = −0.048, p = 0.645). Longer fusions were associated with larger (r = 0.42, P = 0.01) and less flexible (r = −0.37, p = 0.02) MT curves.

The mean postoperative Cobb angle was 18.6 ± 4.2°, giving a mean correction of 72.5 ± 6.8%. The mean absolute change in the Cobb angle after surgery was 49.9 ± 8.3°. The difference in the preoperative and postoperative Cobb angles was significant (*P* < 0.0001). There was a significant correlation between the flexibility of the curve as assessed by push-prone radiographs with the percentage correction achieved (r = 0.368, p < 0.0001) as well as absolute correction in degrees (r = 0.643, p < 0.0001). No significant correlations were present between metal density and MT curve coronal correction rate/percentage (r = 0.086, p = 0.407) or postoperative Cobb angle (r = 0.098, p = 0.344).

The mean preoperative sagittal thoracic kyphosis (T5–T12) was 25.8 ± 6.8° and decreased to 21.6 ± 5.9° after surgery, which was statistically significant (*P* < 0.0001). The difference between preoperative and post-operative lumosaccral lordosis was significant (P < 0.0001). Metal density was not correlated with absolute change in thoracic kyphosis (r = 0.202, p = 0.05) or lumbosacral lordosis (r = 0.013, p = 0.899).

## 3 Discussion

This study evaluates the impact of a limited pedicle screw density on degree of correction achieved in patients with AIS. It demonstrates that constructs with implant densities of approximately 60% can safely be used in moderate, flexible (45–80°, average 47% flexibility) idiopathic curves to attain and maintain correction in AIS. Our data did not show correlation between the metal density and the pre-operative radiographic variables. Thus, although intuition would indicate that larger, stiff MT curves would be accompanied by increasing metal density this was not the case in our study. The percent correction achieved in main thoracic and secondary curves was comparable to past literature [5,28,29].

The primary aims of surgery in AIS are to halt curve progression, reduce the patient's deformity and improve their quality of life [19]. However, from a developing nation's perspective, the debate moves around the most cost-effective method of treatment and is considered primary determinant for feasibility of a procedure. Implants are the greatest contributor to increasing costs in AIS surgery. In their study, Ruston et al. [19] identified that pedicle screws to represent 20–30% of the overall inpatient costs in AIS surgery. Implant cost may represent 50% of total cost when metal density is approximately 80% [30]. In a health-care system where patients themselves are primary payers of medicare services, excessive usage of implants increase the total cost of procedure and beyond the reach of most patients. Our hospital is the first and largest spine center in Pakistan and receives patients from all over the country. It is a welfare trust setup which offers international standard yet subsidized services to most deserving patients with donations and alms making for the cost difference. Patients usually bear cost of implant and medication with subsidy on hospital accommodation, theatre charges, surgeon and anesthetist fee. Even the implants are obtained at bargained prices from companies.

Though surgeons innately wish to improve radiographical and clinical curve correction, it has been difficult to establish strong correlations between radiographical curve correction and clinical outcomes [31], health-related quality of life outcome scores [32], or patient satisfaction [33]. We did not include the results of any patient based health related outcome tool, as these were not collected routinely at the time these patients were operated upon. However, previous studies have failed to demonstrate any strong correlation between curve correction and measurable HRQOL [10,34].

Several authors report that addition of each point of fixation provides broader stress distribution within the instrumented spine, thereby allowing increased curve correction by virtue of greater overall loads being applied to the spine without risking screw pullout [6,8]. Although differentiation between high and low density constructs is arbitrary, high density construct refer to instrumentation of each available pedicle (2 screws in each vertebra) to achieve maximum correction. On the contrary, other studies have demonstrated that lower-density instrumentation such as interval pedicle screw placement constructs is an efficient and safe alternative with similar corrective effects [10,11,23,24,26]. In their randomized prospective study, Li et al. [11] compared consecutive versus interval pedicle screw placement on the concave side in patients with a Lenke Type 1 scoliosis and found no difference with respect to coronal and sagittal plane correction. Quan et al. [5] observed no correlation between implant density and coronal or sagittal correction. They found that increased correction of the main thoracic curve was associated with increased curve flexibility. In a multicenter study, Clements et al. [6] found a significant correlation between implant density and major curve correction at 2 years. The highest amount of correction was observed when bilateral segmental screws were used. However, the authors also observed an inverse correlation between implant density and postoperative kyphosis. The present study supports the findings of previous studies, but includes a broader range of Lenke type curves, larger patient volume, and matched single institution comparison cohort. This is the first and largest study of its kind to be reported from Pakistan.

The biomechanical principle while selecting number of screws or a specific screw configuration is to improve stress distribution [1]. Several low-density screw constructs have been reported in the literature [10,23,26,35] without definitive evidence of superiority of one construct over others [1]. Almost all the variations in low-density constructs include bilateral pedicle screw insertion at uppermost, apical and lowermost instrumented vertebrae with skip pedicle screws in between [1]. In our study, we did not utilize a fixed construct strategy. Pedicle screw location and density was determined by the surgeon intra-operatively based on perceived curve stiffness and fusion length with an even distribution of fixation points along the construct.

In addition to screw density, reduction technique, location of implants, the quality of the facetal release and resection, rod diameter and have been shown to affect outcomes in both the sagittal and coronal planes, but commonly not reported [[6], [7], [8], [9], [10]]. Controlling for rod diameter, Quan and Gibson found no correlation between metal density and coronal/sagittal outcomes using 6 mm titanium rods [5].

This study is limited by short-term patient follow-up. We did not account for future revision operation. The lack of patient reported outcome measures (PROMS) is a weakness of the current study. Our retrospective radiographic study has not compared the complications that metal density may influence. Furthermore, we could not identify a critical level of metal density below which deformity corrections declines.

## 4 Conclusion

We conclude that excellent curve correction, stability, and balance can be achieved using a limited-density pedicle screw construct in the treatment of adolescents with idiopathic scoliosis of moderate severity.

## Ethical approval

This is a retrospective review of medical records which did not compromise patient care. Need for ethical approval was waived by Institutional Review Board (Copy available on demand).

## Sources of funding

No funding or sponsors was used for this study.

## Author contribution

**Irfan Qadir:** Data collection, data analysis, literature review, manuscript writing.

**Abdullah Shah:** Study concept, literature review, manuscript writing, Patient care.

**Syed Roman Alam:** Data collection, manuscript writing.

**Haseeb Hussain:** Data analysis, manuscript writing.

**Rizwan Akram:** Literature review, manuscript writing.

**Amer Aziz:** Literature review, manuscript writing, Patient care.

## Registration of research studies

Name of the registry: clinicaltrial.gov.

Unique Identifying number or registration ID: NCT04175145.

Hyperlink to the registration (must be publicly accessible): https://clinicaltrials.gov/ct2/show/NCT04175145?term=NCT04175145&draw=2&rank=1.

## Guarantor

Irfan Qadir: Correspoding author.

## Provenance and peer review

Not commissioned, externally peer reviewed.

## Declaration of competing interest

Authors declare no conflict of interest.

## References

[1] Le Naveaux F., Aubin C.E., Larson A.N., Polly D.W., Baghdadi Y.M., Labelle H. (2015). Implant distribution in surgically instrumented Lenke 1 adolescent idiopathic scoliosis: does it affect curve correction?. Spine.

[2] Kim Y.J., Lenke L.G., Cho S.K., Bridwell K.H., Sides B., Blanke K. (2004). Comparative analysis of pedicle screw versus hook instrumentation in posterior spinal fusion of adolescent idiopathic scoliosis. Spine.

[3] Liljenqvist U., Hackenberg L., Link T., Halm H. (2001). Pullout strength of pedicle screws versus pedicle and laminar hooks in the thoracic spine. Acta Orthop. Belg..

[4] Suk S.I., Lee C.K., Kim W.J., Chung Y.J., Park Y.B. (1995). Segmental pedicle screw fixation in the treatment of thoracic idiopathic scoliosis. Spine.

[5] Quan G.M., Gibson M.J. (2010). Correction of main thoracic adolescent idiopathic scoliosis using pedicle screw instrumentation: does higher implant density improve correction?. Spine.

[6] Clements D.H., Betz R.R., Newton P.O., Rohmiller M., Marks M.C., Bastrom T. (2009). Correlation of scoliosis curve correction with the number and type of fixation anchors. Spine.

[7] Larson A.N., Polly D.W., Diamond B., Ledonio C., Richards B.S., Emans J.B. (2014). Does higher anchor density result in increased curve correction and improved clinical outcomes in adolescent idiopathic scoliosis?. Spine.

[8] Sanders J.O., Diab M., Richards S.B., Lenke L.G., Johnston C.E., Emans J.B. (2011). Fixation points within the main thoracic curve: does more instrumentation produce greater curve correction and improved results?. Spine.

[9] Yang S., Jones-Quaidoo S.M., Eager M., Griffin J.W., Reddi V., Novicoff W. (2011). Right adolescent idiopathic thoracic curve (Lenke 1 A and B): does cost of instrumentation and implant density improve radiographic and cosmetic parameters?. Eur. Spine J. Off. Pub. Eur. Spine. Soc. Eur. Spinal Deformity. Soc. Eur. Sec. Cervical. Spine. Res. Soc..

[10] Bharucha N.J., Lonner B.S., Auerbach J.D., Kean K.E., Trobisch P.D. (2013). Low-density versus high-density thoracic pedicle screw constructs in adolescent idiopathic scoliosis: do more screws lead to a better outcome?. Spine J. : Off. J. North Am. Spine. Soc..

[11] Li M., Shen Y., Fang X., Ni J., Gu S., Zhu X. (2009). Coronal and sagittal plane correction in patients with Lenke 1 adolescent idiopathic scoliosis: a comparison of consecutive versus interval pedicle screw placement. J. Spinal Disord. Tech..

[12] Tsirikos A.I., Subramanian A.S. (2012). Posterior spinal arthrodesis for adolescent idiopathic scoliosis using pedicle screw instrumentation: does a bilateral or unilateral screw technique affect surgical outcome?. J. Bone Jt. Surg. Br. Vol..

[13] Clement J.L., Chau E., Kimkpe C., Vallade M.J. (2008). Restoration of thoracic kyphosis by posterior instrumentation in adolescent idiopathic scoliosis: comparative radiographic analysis of two methods of reduction. Spine.

[14] Vora V., Crawford A., Babekhir N., Boachie-Adjei O., Lenke L., Peskin M. (2007). A pedicle screw construct gives an enhanced posterior correction of adolescent idiopathic scoliosis when compared with other constructs: myth or reality. Spine.

[15] Fletcher N.D., Hopkins J., McClung A., Browne R., Sucato D.J. (2012). Residual thoracic hypokyphosis after posterior spinal fusion and instrumentation in adolescent idiopathic scoliosis: risk factors and clinical ramifications. Spine.

[16] Morr S., Carrer A., Alvarez-Garcia de Quesada L.I., Rodriguez-Olaverri J.C. (2015). Skipped versus consecutive pedicle screw constructs for correction of Lenke 1 curves. Eur. Spine J.; Off. Pub. Eur. Spine. Soc. Eur. Spinal Deformity. Soc. Eur. Sec. Cervical. Spine. Res. Soc..

[17] Lamerain M., Bachy M., Delpont M., Kabbaj R., Mary P., Vialle R. (2014). CoCr rods provide better frontal correction of adolescent idiopathic scoliosis treated by all-pedicle screw fixation. Eur. Spine J. : Off. Pub. Eur. Spine. Soc. Eur. Spinal Deformity. Soc. Eur. Sec. Cervical. Spine. Res. Soc..

[18] Aubin C.E., Labelle H., Ciolofan O.C. (2007). Variability of spinal instrumentation configurations in adolescent idiopathic scoliosis. Eur. Spine J. Off. Pub. Eur. Spine. Soc. Eur. Spinal Deformity. Soc. Eur. Sec. Cervical. Spine. Res. Soc..

[19] Rushton P.R., Elmalky M., Tikoo A., Basu S., Cole A.A., Grevitt M.P. (2016). The effect of metal density in thoracic adolescent idiopathic scoliosis. Eur. Spine J. : Off. Pub. Eur. Spine. Soc. Eur. Spinal Deformity. Soc. Eur. Sec. Cervical. Spine. Res. Soc..

[20] Di Silvestre M., Bakaloudis G., Lolli F., Vommaro F., Martikos K., Parisini P. (2008). Posterior fusion only for thoracic adolescent idiopathic scoliosis of more than 80 degrees: pedicle screws versus hybrid instrumentation. Eur. Spine J. Off. Pub. Eur. Spine. Soc. Eur. Spinal Deformity. Soc. Eur. Sec. Cervical. Spine. Res. Soc..

[21] Fu G., Kawakami N., Goto M., Tsuji T., Ohara T., Imagama S. (2009). Comparison of vertebral rotation corrected by different techniques and anchors in surgical treatment of adolescent thoracic idiopathic scoliosis. J. Spinal Disord. Tech..

[22] Kemppainen J.W., Morscher M.A., Gothard M.D., Adamczyk M.J., Ritzman T.F. (2016). Evaluation of limited screw density pedicle screw constructs in posterior fusions for adolescent idiopathic scoliosis. Spine deformity.

[23] Hwang C.J., Lee C.K., Chang B.S., Kim M.S., Yeom J.S., Choi J.M. (2011). Minimum 5-year follow-up results of skipped pedicle screw fixation for flexible idiopathic scoliosis. J. Neurosurg. Spine.

[24] Min K., Sdzuy C., Farshad M. (2013). Posterior correction of thoracic adolescent idiopathic scoliosis with pedicle screw instrumentation: results of 48 patients with minimal 10-year follow-up. Eur. Spine J. Off. Pub. Eur. Spine. Soc. Eur. Spinal Deformity. Soc. Eur. Sec. Cervical. Spine. Res. Soc..

[25] Takahashi J., Ikegami S., Kuraishi S., Shimizu M., Futatsugi T., Kato H. (2014). Skip pedicle screw fixation combined with Ponte osteotomy for adolescent idiopathic scoliosis. Eur. Spine J. Off. Pub. Eur. Spine. Soc. Eur. Spinal Deformity. Soc. Eur. Sec. Cervical. Spine. Res. Soc..

[26] Li J., Cheung K.M., Samartzis D., Ganal-Antonio A.K., Zhu X., Li M. (2016). Key-Vertebral screws strategy for main thoracic curve correction in patients with adolescent idiopathic scoliosis. Clinical spine surgery.

[27] Agha R.A., Borrelli M.R., Vella-Baldacchino M., Thavayogan R., Orgill D.P. (2017). The STROCSS statement: strengthening the reporting of cohort studies in surgery. Int. J. Surg..

[28] Suk S.I., Lee S.M., Chung E.R., Kim J.H., Kim S.S. (2005). Selective thoracic fusion with segmental pedicle screw fixation in the treatment of thoracic idiopathic scoliosis: more than 5-year follow-up. Spine.

[29] Kuklo T.R., Potter B.K., Polly D.W., Lenke L.G. (2005). Monaxial versus multiaxial thoracic pedicle screws in the correction of adolescent idiopathic scoliosis. Spine.

[30] Martin C.T., Pugely A.J., Gao Y., Mendoza-Lattes S.A., Ilgenfritz R.M., Callaghan J.J. (2014). Increasing hospital charges for adolescent idiopathic scoliosis in the United States. Spine.

[31] Danielsson A.J. (2007). What impact does spinal deformity correction for adolescent idiopathic scoliosis make on quality of life?. Spine.

[32] Howard A., Donaldson S., Hedden D., Stephens D., Alman B., Wright J. (2007). Improvement in quality of life following surgery for adolescent idiopathic scoliosis. Spine.

[33] Smith P.L., Donaldson S., Hedden D., Alman B., Howard A., Stephens D. (2006). Parents' and patients' perceptions of postoperative appearance in adolescent idiopathic scoliosis. Spine.

[34] Kim Y.J., Lenke L.G., Kim J., Bridwell K.H., Cho S.K., Cheh G. (2006). Comparative analysis of pedicle screw versus hybrid instrumentation in posterior spinal fusion of adolescent idiopathic scoliosis. Spine.

[35] Gotfryd A.O., Avanzi O. (2013). Randomized clinical study on surgical techniques with different pedicle screw densities in the treatment of adolescent idiopathic scoliosis types Lenke 1A and 1B. Spine deformity.

